# Peripheral Injection of Tim-3 Antibody Attenuates VSV Encephalitis by Enhancing MHC-I Presentation

**DOI:** 10.3389/fimmu.2021.667478

**Published:** 2021-05-07

**Authors:** Ge Li, Lili Tang, Chunmei Hou, Zhiding Wang, Yang Gao, Shuaijie Dou, Rongliang Mo, Ying Hao, Zhenfang Gao, Yuxiang Li, Jie Dong, Jiyan Zhang, Beifen Shen, Renxi Wang, Gencheng Han

**Affiliations:** ^1^ Department of Neuroimmune and Antibody Engineering, Beijing Institute of Basic Medical Sciences, Beijing, China; ^2^ Beijing Institute of Brain Disorders, Laboratory of Brain Disorders, Ministry of Science and Technology, Collaborative Innovation Center for Brain Disorders, Capital Medical University, Beijing, China

**Keywords:** encephalitis, vesicular stomatitis virus, Tim-3, macrophages, major histocompatibility complex-I, ubiquitination

## Abstract

Viral encephalitis is the most common cause of encephalitis. It is responsible for high morbidity rates, permanent neurological sequelae, and even high mortality rates. The host immune response plays a critical role in preventing or clearing invading pathogens, especially when effective antiviral treatment is lacking. However, due to blockade of the blood-brain barrier, it remains unclear how peripheral immune cells contribute to the fight against intracerebral viruses. Here, we report that peripheral injection of an antibody against human Tim-3, an immune checkpoint inhibitor widely expressed on immune cells, markedly attenuated vesicular stomatitis virus (VSV) encephalitis, marked by decreased mortality and improved neuroethology in mice. Peripheral injection of Tim-3 antibody enhanced the recruitment of immune cells to the brain, increased the expression of major histocompatibility complex-I (MHC-I) on macrophages, and as a result, promoted the activation of VSV-specific CD8^+^ T cells. Depletion of macrophages abolished the peripheral injection-mediated protection against VSV encephalitis. Notably, for the first time, we found a novel post-translational modification of MHC-I by Tim-3, wherein, by enhancing the expression of MARCH9, Tim-3 promoted the proteasome-dependent degradation of MHC-I *via* K48-linked ubiquitination in macrophages. These results provide insights into the immune response against intracranial infections; thus, manipulating the peripheral immune cells with Tim-3 antibody to fight viruses in the brain may have potential applications for combating viral encephalitis.

## Introduction

Viral encephalitis is defined as a pathological inflammation of the brain parenchyma secondary to viral infection. Worldwide, rabies and Japanese encephalitis viruses are responsible for an estimated annual mortality of 60,000 and 17,000 people, respectively (1, 2). Encephalitis is associated with appreciable mortality and high rates of permanent neurological impairment in survivors, and in most cases, there is no available antiviral therapy. Therefore, insight into its pathogenesis is urgently needed (3). Encephalitis may result from direct viral exposure, hematogenous spread, or retrograde infection of the nervous system. Meanwhile, limited vaccine availability and efficiency challenge the prevention of viral encephalitis (4). Innate immune cells, including resident microglia, recruited peripheral monocyte-derived macrophages (pMDM), and recruited peripheral T cells, play critical roles in viral encephalitis prevention or clearance (5). However, the mechanisms by which resident cells cooperate with the peripheral immune cells to fight viral encephalitis are still undetermined.

Central nervous system (CNS) is an immune-privileged organ towing to the presence of the blood-brain barrier (BBB) and relatively low number of surveilling peripheral immune cells within the brain parenchyma (6). However, during neuroinfection or chronic neuroinflammation, peripheral immune cells infiltrate the CNS and accumulate near sites of infection (7). Microglia are the CNS-resident mononuclear phagocytic cells that are typified by a unique ramified shape and distinctive gene expression (8). Unlike blood-derived macrophages, most microglia are derived from a yolk sac progenitor that seed the brain during early development (9, 10). Recent studies suggest that microglia are important for neurogenesis, synapse maintenance, neuroimmune homeostasis, and neuronal function, thereby indicating that these cells maintain a healthy brain by performing a multitude of functions (11–15). During viral encephalitis, the absence of microglia leads to more rapid viral replication, which allow the virus to evade the immune response. Microglia are reportedly required between days 0 and 6 post-infection with depletion at later stages having no effect on survival, thereby suggesting that microglia play a role predominantly in the early stages of infection. As a consequence of cytokine and chemokine secretion, viral infection of the CNS results in recruitment of innate and adaptive immune cells to the brain. Virus-specific CD8^+^ T cells, which are detectable within the brain 5–7 days post-infection, are critical for viral clearance (16, 17). Spontaneous recruitment of peripheral immune cells seems to be insufficient under neuroinflammation or neuroinfection (18); thus, it is of great interest to identify mechanisms of recruitment and methods of manipulating this process.

In recent years, immune checkpoint inhibitors, such as programmed cell death protein-1 (PD-1), cytotoxic T-lymphocyte-associated protein 4 (CTLA-4), and T cell immunoglobulin and mucin domain-containing protein 3 (Tim-3), have attracted considerable attention as these molecules play critical roles in maintaining immune homeostasis, and their dysregulation is associated with many immune-related diseases, tumor development, chronic infections, and autoimmune diseases. Antagonists against PD-1, which can systematically enhance the immune response, are effective in many tumors. Interestingly, a report showed that enhanced systemic immune response induced by a PD-1 antagonist may also have a therapeutic role in Alzheimer’s disease (19, 20). Although the underlying mechanisms remain to be determined, the data suggest that immune checkpoint molecules may be widely targeted in many immune-related disorders.

Tim-3 was initially identified on activated Th1, Th17, and Tc1 cells, and engagement of Tim-3 reportedly leads to T cell tolerance or failure (21). It was later observed that Tim-3 is also expressed on innate immune cells such as macrophages (22). Tim-3 plays a critical role in maintaining the tolerance of macrophages in both tumors and infectious diseases (23, 24). Compared with PD-1, which is mainly expressed on T cells, the wider distribution of Tim-3 implies that it may play crucial roles in modulating the systemic immune response. Thus, it is of great interest whether peripheral injection of Tim-3 antagonist may enhance immune response systematically, and whether it can be helpful in fighting against neuroinfection.

Here, we employed a vesicular stomatitis virus (VSV) encephalitis mouse model to investigate the mechanism by which innate and adaptive immune cells thwart neuroinvasion with or without modulation of the peripheral immune response by Tim-3 antagonist. Our data showed that peripheral injection of Tim-3 antibody significantly enhanced the migration and antigen-presenting activity of macrophages, which contributed to virus control in viral encephalitis.

## Materials and Methods

### Mice

Male C57BL/6 mice (6–8 weeks old) were purchased from Beijing Vital River Laboratory Animal Technology Co. Ltd., China. All mice were bred and maintained under specific pathogen-free conditions, and the experimental protocol was approved by the Ethics Committee of Animal Experiments of the Institute of Military Cognitive and Brain Sciences (permit number: AMMS2020-0166). All efforts were made to minimize animal suffering.

### Cell Culture and Transfection

RAW264.7, Vero, and HEK293T cells (American Type Cell Culture, Manassas, VA, USA) were maintained in Dulbecco’s Modified Eagle’s Medium (DMEM; Gibco, USA) supplemented with 10% fetal bovine serum (FBS; Gibco, USA). Mouse peritoneal macrophages were collected as previously described (24). For transient expression, the vector (pcDNA3.1) alone, plasmids coding full-length human Tim-3 (Tim-3-pcDNA3.1), or plasmids coding membrane associated ring-CH-type finger-9 (MARCH9) were transfected into HEK293T cells for 48 h.

### *In Vivo* Experimental VSV Infection

VSV was a gift from Prof. Minghong Jiang at the Institute of Basic Medicine, Chinese Academy of Medical Sciences. VSV was cultivated as previously described (25). Mice were anesthetized by intraperitoneal injection of pentobarbital (150 mg/kg). Intracranial injections were performed on the left side, 1.5 mm lateral and 2.0 mm rostral of the bregma at 2.0 mm depth using micro-syringes from Gaoge (Shanghai, China) controlled by a stereotactic injector from Longer (Shanghai, China). Next, 2 µL VSV was injected at a concentration of 1 × 10^6^ pfu/g for 10 min and the needle was kept in place for an additional 10 min before removal.

The monoclonal antibody against human Tim-3 (clone A3) was originally obtained by screening human natural phage antibodies library using recombinant human Tim-3 protein as bait. To test the efficacy of anti-Tim-3 antibody in VSV infection, mice were injected intraperitoneally with 10 mg/kg of neutralizing antibody specific for Tim-3 or human IgG1 isotype control antibody (BioLegend, USA) diluted in 200 µL of phosphate-buffered saline (PBS) both 48 h and 24 h before and after injection with VSV.

### Quantification of Viral Load

VSV load in brain tissue samples was determined by TCID50 assay (50% tissue culture infectious dose), which is a method to measure the amount of infectious virus in a sample by determining the highest dilution of the sample that can infect 50% of the cells in a culture. Virus mRNA replication was analyzed by reverse transcriptase quantitative-polymerase chain reaction (RT-PCR (26);. Brain tissues were collected on day 5 after infection, transferred to lysing matrix tubes, and incubated in 1000 µL DMEM (10% FBS). Serial 10-fold dilutions of supernatant were added to Vero cell monolayers in 96-well plates and they were incubated for 72 h at 37°C. Endpoints of cytopathic effect were observed, and TCID50 was determined using the Reed-Muench method. For RT-PCR, samples were subjected to RNA extraction and cDNA synthesis, as described previously. Then, cDNA was amplified using SYBR Green I Master Mix (Roche, Basel, Switzerland) and a LightCycler 480 PCR system (Roche) with primers targeting the VSV gene (forward primer: 5′-CAAGTCAAAATGCCCAAGAGTCACA-3′ and reverse primer: 5′-TTTCCTTGCATTGTTCTACAGATGG-3′). Results are expressed as the relative number of genome copies of VSV per sample.

### Behavioral Assessment

Behavioral changes in mice following VSV infection were recorded using the open-field test (OFT) and automated computer-assisted method (CatWalk, Noldus Information Technology Inc., Netherlands). The OFT was used to examine both locomotor activity and anxious behavior. The floor of the open field was divided into 16 equal rectangles using black lines, wherein set area was zone1 and the rest of the rectangles were zone2. Fusion software (ANY-maze) analyzed various parameters based on recorded activity, including total distance, time in zone1, and average duration of visit to zone1. Each mouse was individually placed in the middle of the apparatus and allowed to explore for 2 min. Animals were tested twice on consecutive days on the OFT to examine habituation.

Gait analysis was performed on mice that could walk using the CatWalk system. Five trials per mouse, with a maximum of 10 s to traverse the glass plate, were performed. The gait analysis system is designed to dynamically measure the footprints of moving animals and assess any locomotor deficits in animal models; these are widely used in viral encephalitis studies. The regularity index (%) is a fractional measure of inter-paw coordination, which expresses the number of normal step sequence patterns relative to the total number of paw placements. In healthy, fully coordinated animals, the regularity index is close to 100%. Swing speed provides the speed of the paws during the swing phase. Meanwhile, stride length characterizes the distance between the placement of the paw and subsequent placement of the same paw. These data were analyzed using the Catwalk software.

### Flow Cytometry

Brain tissues were excised from mice and individually homogenized. Then, the tissues were incubated with 1 mg/mL collagenase D (Roche) and 15 µg/mL DNase (Invitrogen, USA) for 40 min at 37°C, filtered through a 70 μM filter, suspended in 40% Percoll (GE Healthcare, USA), in RPMI 1640, overlayered with 70% Percoll, and centrifuged for 20 min at 4°C and 2000 rpm. Cells at the 40%–70% interface were collected, diluted in PBS, centrifuged for 8 min at 1500 rpm, and resuspended as a single-cell suspension for analysis. Spleens were dissected and scrubbed through a 70 µM nylon mesh cell strainer, then subjected to density gradient centrifugation (Ficoll-Hypaque, TBD Science, Tianjin, China) for 20 min at 24°C and 2000 rpm. The cells at the Ficoll interface were collected, diluted in PBS, and centrifuged for 8 min at 1500 rpm. The cells were then resuspended in flow cytometry wash buffer (2% FBS in PBS) for staining according to standard protocols using the following antibodies: CD11b-Percp, CD45-FITC, CD4-APC, and CD8-BV510 (Biolegend, San Diego, CA, USA). The gating strategy employed to quantify frequencies of infiltrating and resident immune cells is as described previously (5). Total immune cells were initially gated based on expression of the leukocyte common antigen CD45 and side scatter, and further gated by CD11b expression to distinguish microglia (CD11b^+^CD45^Low^) (P1) from non-myeloid infiltrating lymphocytes (CD11b^-^CD45^hi^) (P3) and infiltrating monocytes derived macrophages (CD11b^+^CD45^hi^) (P2) (27). Tetramer staining of brain and spleen cells was performed using a PE-conjugated MHC-I (H2K^b^) tetramer folded with the VSV epitope peptide RGYVYQGL (MBL, Japan (28);. Data were acquired on a FACSCalibur flow cytometer (BD Biosciences, USA) and analyzed using Flow Jo software.

### Western Blot Analysis and Co-immunoprecipitation

For the western blot analysis, cells were harvested and lysed in lysis buffer supplemented with protease and phosphatase inhibitors (Sigma Aldrich). Samples were separated by 10% sodium dodecyl sulfate-polyacrylamide gel electrophoresis (SDS-PAGE), and the expression of the indicated proteins with the indicated antibodies was examined.

For co-immunoprecipitation, cells were collected 24 h after transfection and lysed in lysis buffer supplemented with a protease inhibitor cocktail. After centrifugation for 15 min at 12 000 rpm and 4°C, the supernatant was collected and incubated with Protein A/G Sepharose beads (SC-2003, Santa Cruz) coupled to specific antibodies overnight with slow rotation at 4°C. The next day, beads were washed three times with high-salt wash buffer and three times with low-salt wash buffer, and finally eluted by boiling for 10 min with 5× sample buffer (as indicated). Precipitates were fractionated using SDS-PAGE at appropriate concentrations (as indicated).

### Ubiquitination Assay

To analyze the ubiquitination of the major histocompatibility complex-I (MHC-I) in HEK293T cells, plasmids encoding ubiquitin with HA tag (HA-ubiquitin), or Ubiquitin K48 with HA tag (Ub-k48) which mediates proteasomal-dependent degradation of the target protein, were transfected into HEK293T cells for 24 h and then treated with 20 μM MG132, a proteosome inhibitor, for 6 h before harvesting. Cells were lysed with immunoprecipitation lysis buffer (1% NP-40, 20 mM; Tris-HCl, 150 mM; NaCl, 5 mM; EDTA, 1 mM; Na_3_VO_4_, 0.5%; and sodium deoxycholic acid and complete protease inhibitor cocktail [Roche], pH 7.5), and then whole-cell lysates were immunoprecipitated with antibodies against Flag-tag (F1804), followed by analysis of ubiquitination of MHC-I with MHC-I antibody (Proteintech,15240-1-AP). Precipitates were fractionated using SDS-PAGE at appropriate concentrations (as indicated).

### Real-Time Quantitative RT-PCR

Total RNA was extracted using TRIzol reagent by following the manufacturer’s instructions. Real-time quantitative RT-PCR analysis was performed using SYBR Green I Master Mix (Roche, Basel, Switzerland) and a Light Cycler 480 PCR system (Roche). The relative expression of the gene of interest was determined using the 2^–ΔΔCt^ method, with 18S ribosomal mRNA (18S) used as the internal control. Primers used for RT-PCR are listed in **Table S1**.

### Histopathology and Immunofluorescence

Brains were post-fixed overnight with 4% formaldehyde after their removal from the mice. Sections were made in a rostral to caudal fashion, and were mounted and stained with hematoxylin and eosin. Sections were analyzed for brain pathology and signs of inflammation, focusing on the regions of the cortex/meninges. For immunofluorescence, anti-MHC-I-VSV-tetramers and anti-CD8 antibodies were used (MBL,TS-M529-1). Images were obtained at 200–400× magnification using an Olympus BX51 optical microscope equipped with a camera.

### Statistical Analysis

Statistical significance was determined using a one-way ANOVA followed by a two-tailed Student’s *t*-test. For the mouse survival study, Kaplan-Meier survival curves were generated and analyzed for significance using GraphPad Prism 8.0.A P value <0.05 was considered statistically significant.

## Results

### Peripheral Injection of Tim-3 Antibody Attenuates VSV Encephalitis in Mice

**Figure 1:**
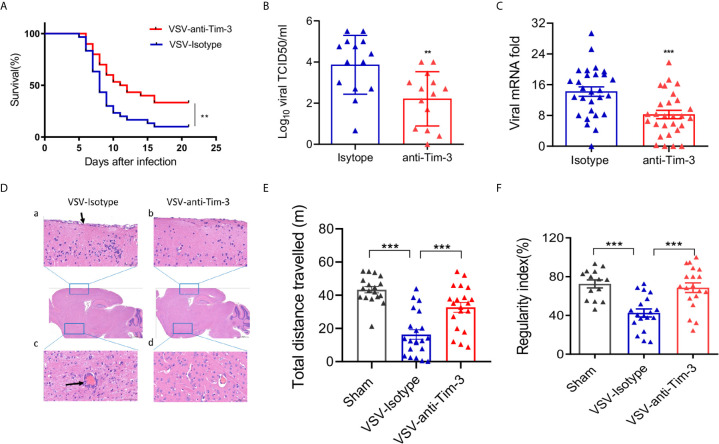
Peripheral injection of Tim-3 antibody attenuates VSV encephalitis in mice. **(A)** C57BL/6 mice were intracranially injected with VSV (1 × 10^6^ pfu/g) on day 0 and intraperitoneally injected with Tim-3 antibody (10 mg/kg, n=15) or isotype control (n=15) on days -2,-1, 1, and 2. Survival rate was analyzed. **(B–D)** Mice were infected and treated as elaborated in **(A)**. Brain tissues were collected on day 5 post-infection and VSV loads were analyzed by TCID50 assay (B, n=14 in each group). VSV replication were analyzed by real-time polymerase chain reaction (C, n=28 in each group), and **(D)** Hematoxylin and eosin stain of the brain section. Arrow in a) shows the damaged meningitis which is absent in b). Arrow in c) shows perivascular lymphocyte cuffing which is a well-known indicator of inflammation in the brain. No perivascular lymphocyte cuffing was found in d). The data shown are representative of six samples from each group. Original magnification ×100. **(E)** Mice underwent the open-field test, and their spontaneous locomotor activity was evaluated by measuring the distance traveled in a defined area for 10 min. **(F)** Mice underwent the CatWalk analysis assay to evaluate locomotor deficits *via* measuring the regularity index with a maximum of 10 s permissible to traverse a glass plate. In **(B, C, E, F)**, the data are expressed as mean ± SEM of three independent experiments. **p < 0.01, ***p < 0.001.

To determine whether manipulating the peripheral immune response affects the outcome of VSV encephalitis, a monoclonal antibody against human Tim-3 (clone A3) was injected into mice on days -2, 0, and 2 of intracerebral VSV injection. This antibody of Tim-3 binds to both human and mouse Tim-3 (**Figures S1A, B**) and showed excellent neutralizing activity in human and mouse macrophage cell lines *in vitro* (**Figures S1C, D**) and in healthy mice *in vivo* (**Figure S2**). Peripheral injection of Tim-3 antibody increased the survival of mice with VSV encephalitis than the isotype control mice (**Figure 1A**). We then examined the viral load, viral replication, and tissue damage of the brain tissues acquired from mice with VSV encephalitis, with or without Tim-3 antibody injection. **Figures 1B, C** show that peripheral injection of Tim-3 antibody inhibited viral replication and attenuated tissue damage. **Figure 1D** shows that Tim-3 antibody injection markedly decreased meningeal injury and perivascular cuffing. Finally, we examined the effects of Tim-3 antibody injection on the neuroethology of mice using open-field test (OFT) and CatWalk assay. The data showed that mice that received Tim-3 antibody moved a longer distance in the open field (**Figure 1E**) and showed a higher regularity index (**Figure 1F**) than mice that received the control antibody. Spontaneous locomotor activity and locomotor capacity were also improved by Tim-3 antibody injection (**Figure S3**). These data showed that peripheral injection of Tim-3 antibody inhibited viral replication and attenuated the symptoms of VSV encephalitis.

### Peripheral Injection of Tim-3 Antibody Enhances the Recruitment of pMDM and T Cells Into the Brain

**Figure 2:**
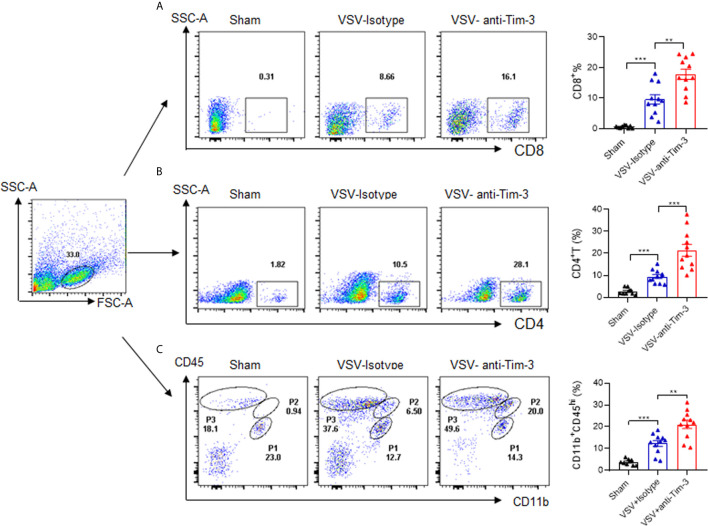
Peripheral injection of Tim-3 antibody enhances the recruitment of peripheral immune cells. Mice were treated as shown in **Figure 1**. On day 5 post-infection, mice were sacrificed, brain tissues were collected from them, and single-cell suspensions were prepared as described in the Material and Methods. The infiltration of CD4^+^T cells **(A)**, CD8^+^T cells **(B)** and peripheral monocyte-derived macrophages (pMDM) cells (CD11b^+^CD45^hi^) **(C)** were examined by flow cytometry analysis. The left panel shows the representative fluorescence-activated cell sorting (FACS) plot, and the right panel are presented as means ± SEM of three independent experiments. **p < 0.01, ***p < 0.001.

Because of cytokine and chemokine secretion, viral infection of the CNS may result in the recruitment of innate and adaptive immune cells to the brain. To determine the mechanisms of Tim-3 antibody-mediated protection against VSV encephalitis, we first examined whether there was enhanced immune cell recruitment following antibody injection. The data in **Figure 2** show that CD8^+^ T cells, CD4^+^ T cells, and CD11b^+^CD45^hi^ cells, which are pMDM, were all markedly increased. The recruitment of peripheral immune cells to the brain is associated with the induction of chemokines (29). We also examined the expression of chemokines within the brain and chemokine receptors on T cells and macrophages in the presence or absence of Tim-3 antibody injection. Our data showed that peripheral injection of Tim-3 antibody increased the expression of C-C motif chemokine ligand 3 (CCL3) and C-C motif chemokine ligand 5 (CCL5) within the brain, and of C-C chemokine receptor type 5 (CCR5) and C-X-C chemokine receptor 3 (CXCR3) on the macrophages and CD8^+^ T cells, respectively (**Figure S4**). These data showed that Tim-3 antibody injection enhanced the recruitment of peripheral immune cells by increasing chemokine/chemokine receptor expression.

### Depletion of Macrophages Abolished Tim-3 Antibody-Mediated Protection Against VSV Encephalitis

**Figure 3:**
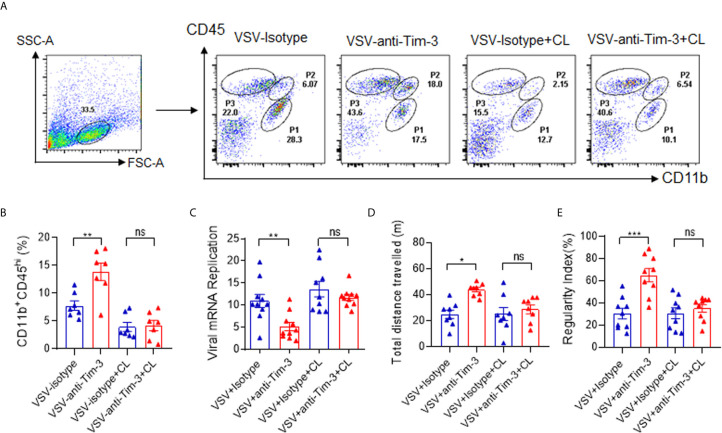
Deletion of macrophages abolished Tim-3 antibody-mediated immune protection against VSV encephalitis. Mice were treated as shown in **Figure 1**, and untreated mice were used as sham controls. For macrophage depletion, Clodronate Liposomes (CL) were intravenously injected three times on days -2, 0, and 2. On day 5 post-infection, the mice were sacrificed. Brain tissues were collected, and single-cell suspensions were prepared and stained for detecting CD11b and CD45 expression by flow cytometry analysis. **(A)** shows the representative fluorescence-activated cell sorting (FACS) plot; while, **(B)** shows the mean ± SEM of three independent experiments. **(C)** The brain tissues were analyzed for VSV replication by real-time polymerase chain reaction, and the results are expressed as mean ± SEM of three independent experiments. **(D)** Mice were subjected to the open-field test to evaluate spontaneous locomotor activity by measuring the distance traveled in a defined area for 10 min. Data are expressed as mean ± SEM of three independent experiments. **(E)** Mice were subjected to the CatWalk analysis to evaluate locomotor deficits by measuring the regularity index, with maximum 10 s to traverse a glass plate. Data are expressed as mean ± SEM of three independent experiments. ns, not significant, *p < 0.05, **p < 0.01, ***p < 0.001.

We have previously found that Tim-3 blockade enhances macrophage activation (23, 24), and another study has shown that peripheral monocytes/macrophages play a protective role in the immune response against viral encephalitis (30). To determine whether macrophages account for Tim-3 antibody-mediated protection against VSV encephalitis, Clodronate Liposomes were used to deplete the monocytes/macrophages. In murine spleen, Clodronate Liposomes had an efficient depletion on macrophages (**Figures S6A, B**). In **Figures 3A, B** show that when macrophages were depleted, VSV infection-induced and Tim-3 antibody-enhanced macrophage recruitment disappeared. More importantly, macrophage depletion also abolished Tim-3 antibody-mediated suppression of virus replication (**Figure 3C**). Tim-3 antibody improved neuroethology as evidenced by comparable locomotor activity (**Figure 3D**) and locomotor deficits (**Figure 3E**) than that in the isotype control group. These data suggest that macrophages play a critical role in Tim-3 antibody-mediated protection against VSV encephalitis.

### Peripheral Injection of Tim-3 Antibody Promotes the Activation of VSV-Specific CD8^+^ T Cells and Enhances the Expression of MHC-I on Macrophages/Microglia

**Figure 4:**
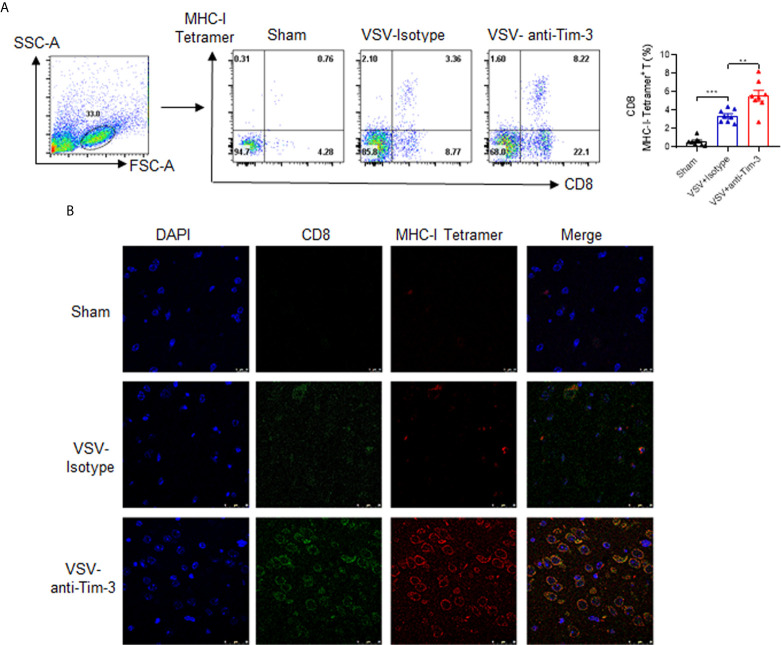
Peripheral injection of Tim-3 antibody enhances the activation of VSV-specific CD8^+^ T cells. Mice were treated as described in **Figure 1**. On day 5 post-infection, brain tissues were collected. **(A)** Single-cell suspensions were prepared and stained with antibodies against CD8 and MHC-I-VSV-tetramer. Left panel shows the representative fluorescence-activated cell sorting (FACS) plot and right panel shows the mean percentage ± SEM of three independent experiments. **P < 0.01, ***P < 0.001. **(B)** The brain tissues were stained with immunofluorescence labels for CD8 (green), MHC-I-VSV-tetramer (red), and DAPI (blue). Data shown are representative images of three independent experiments.

Virus-specific CD8^+^ T cell activation plays a critical role in antiviral immunity. Here, we first examined whether Tim-3 antibody injection promoted VSV-specific CD8^+^ T cell activation by using VSV-peptide/MHC-I tetramer staining in a flow cytometry assay. The data in **Figure 4A** show that the percentage of VSV-specific CD8^+^ T cells was markedly increased within the brain tissues from Tim-3 antibody-treated mice than in those from the control mice. In addition, when the brain tissues were stained with immunofluorescent labels of CD8, MHC-I-VSV-tetramer, and 4′,6-diamidino-2-phenylindole (DAPI), the data confirmed the enhanced VSV-specific CD8^+^ T cell activation in Tim-3 antibody-treated mice (**Figure 4B**).

**Figure 5:**
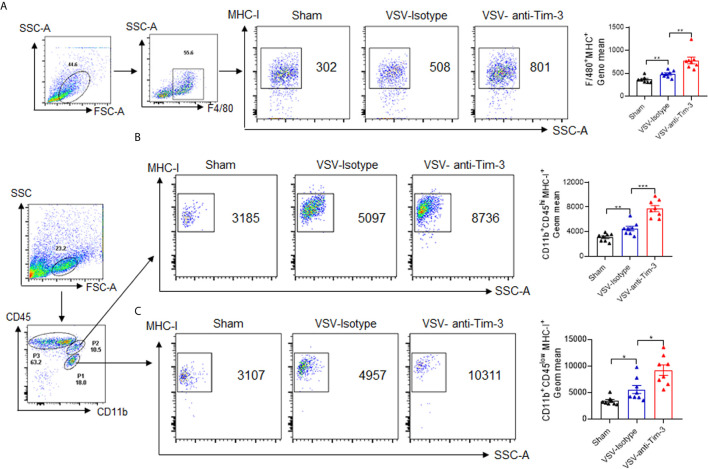
Peripheral injection of Tim-3 antibody enhances the expression of major histocompatibility complex-I (MHC-I). Mice were treated as shown in **Figure 1**. On day 5 post-infection, the mice were sacrificed. **(A)** Peritoneal macrophages were collected and stained with antibodies against F4/80 and MHC-I. The left panel shows the representative fluorescence-activated cell sorting (FACS) plot and right panel shows the mean fluorescence intensity (MFI) ± SEM of three independent experiments. **(B, C)** Brain tissues were collected, and single-cell suspensions were prepared and stained with anti-CD11b and anti-CD45 antibody. CD11b^+^CD45^hi^ pMDM (P2) and CD11b^+^CD45^Low^ microglia (P1) were further gated to analyze the fluorescence intensity of MHC-I using MHC-I antibody. The left panel shows the representative FACS plots and the right panel shows the MFI ± SEM of three independent experiments. *p < 0.05, **p < 0.01, ***p < 0.001.

MHC-I-mediated viral antigen presentation is a key process involved in the activation of CD8^+^ T cells. Many viruses escape immune attack by restricting the surface expression and half-life of MHC-I (31), which reflects the critical role of MHC-I in antiviral immunity. To determine the mechanism by which Tim-3 antibody enhanced CD8^+^ T cell activation, we examined the expression of MHC-I on peripheral and recruited macrophages following Tim-3 antibody injection. The data in **Figure 5** show that the median fluorescence intensity of MHC-I was upregulated on F4/80^+^ peripheral macrophages, CD11b^+^CD45^hi^ pMDM, and CD11b^+^CD45^Low^ microglia in mice that received Tim-3 antibody. These data suggested that Tim-3 antibody enhanced antiviral immunity by promoting MHC-I-mediated antigen presentation and VSV-specific CD8^+^ T cell activation.

### Tim-3 Blockade Increases MHC-I Expression in Macrophages by Inhibiting MARCH9-Mediated Proteasomal Degradation of MHC-I

**Figure 6:**
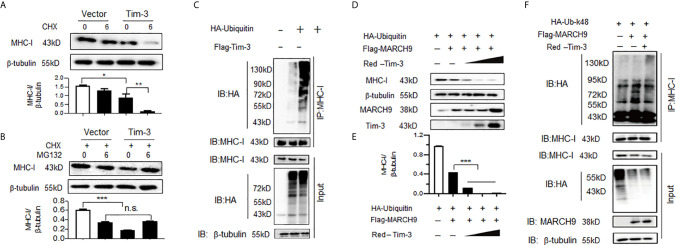
Tim-3 promotes proteasome-dependent degradation and ubiquitination of major histocompatibility complex-I (MHC-I). **(A)** The HEK293T cells were transfected with plasmids encoding Flag-Tim-3 or vector control. Cells were then treated with or without a protein synthesis inhibitor CHX (10 µM) for 6h. Twenty-four hours following transfection, cells were harvested and the level of MHC-I protein was examined by western blot. The upper panel shows the representative image of three independent experiments and the down panel shows the value of the MHC-I/β-tubulin protein ratio which is expressed as mean ± SEM of three independent experiments. **(B)** The HEK293T cells were transfected with plasmids encoding Flag-Tim-3 or vector control. All cells were treated with CHX (10 µM) in the presence or absence of the proteasome inhibitor MG132 (20 µg/ml) for 6h. Twenty-four hours following transfection, cells were harvested and the level of MHC-I protein was examined by western blot. The upper panel shows the representative image of three independent experiments and the down panel shows the value of the MHC-I/β-tubulin protein ratio which is expressed as mean ± SEM of three independent experiments. **(C)** Plasmid encoding HA-Tim-3 was transfected into HEK293T cells for 24h, followed by immunoprecipitation with antibody to MHC-I and then Western blot analysis of MHC-I ubiquitination with antibody to HA. The data shown are the representative image for those of three independent experiments. (D&E) The HEK293T cells were transfected with plasmids encoding HA-ubiquitin, Flag-MARCH9 and increasing doses of a plasmid encoding Red-Tim-3 (0.5,1, and 2 µg). The level of MHC-I protein was examined in cells harvested 24h later. D) shows the representative image of three independent experiments and E) shows the value of the MHC-I/β-tubulin protein ratio which is expressed as mean ± SEM of three independent experiments. **(F)** Plasmids encoding HA-tag-Ubiquitin K48 (K48-lined ubiquitination leads to proteasomal-mediated degradation of the target protein), Flag-MARCH9, and Red-Tim-3 were transfected into HEK293T cells. Cells were treated with MG132 (20 µg/ml) for 6h before harvesting protein lysates, followed by immunoprecipitation with antibody to MHC-I and then Western Blot analysis of K48-linked MHC-I ubiquitination with antibody to HA. These experiments were repeated 3 times with similar results. In A&B, the data shown are expressed as mean ± SEM of three independent experiments. **p<0.01; ***p<0.001.

We then investigated the mechanism by which Tim-3 blockade enhanced MHC-I expression. Surface expression and half-life of MHC-I (the molecule responsible for CD8^+^ T cell antigen presentation and initiation of adaptive immunity) are controlled *via* ubiquitination. Here, we observed for the first time that Tim-3 was involved in the post-translational modification of MHC-I. The data in **Figures 6A, B** show that when Tim-3 was overexpressed, blocking protein synthesis by CHX led to a decreased MHC-I protein expression; whereas, addition of MG132, a proteasome inhibitor, abolished Tim-3 overexpression-mediated MHC-I decrease. These data show that Tim-3 promoted proteasome-dependent degradation of MHC-I.

Ubiquitination is one of the most versatile post-translational modifications that is indispensable for antiviral immunity, and ubiquitination of MHC-I has been widely investigated recently (32). Next, we tested whether Tim-3 promoted MHC-I degradation *via* ubiquitination modification. The data in **Figure 6C** show that co-transfection with Tim-3 significantly enhanced the ubiquitination of MHC-I. Next, possible E3 ligases were identified. Several MARCH family proteins, including MARCH2, MARCH9, and MARCH10 have been found to be associated with the ubiquitination modification of MHC-I. We first examined whether Tim-3 regulated the protein expression of MARCH. The results showed that in RAW264.7 cells silenced with Tim-3 or treated with Tim-3 blocking antibody, the expression of MARCH9 was decreased (**Figure S5**). Other MARCH proteins, including MARCH2 and MARCH10, were not significantly altered when Tim-3 signaling was blocked (data not shown). To test whether MARCH9 was involved in Tim-3-mediated ubiquitination and degradation of MHC-I, we co-transfected K48-linked ubiquitin, MARCH9, and Tim-3 into HEK293T cells. The data in **Figures 6D, E** show that protein levels of MHC-I were significantly decreased in the presence of MARCH9 and increased dose of Tim-3. We then examined which kind of ubiquitination was involved in Tim-3 mediated MHC-I degradation. As K48- linked ubiquitination leads to proteasomal dependent degradation (33), to test whether Tim-3 enhances the K48-linked ubiquitination, here we co-transfected plasmids encoding ubiquitin-K48, MARCH9 and Tim-3 into HEK293T cells. The data in **Figure 6F** showed a marked increase in MHC-I ubiquitination in the presence of MARCH9 and Tim-3. These data showed that Tim-3 signaling promotes proteasomal degradation of MHC-I *via* MARCH9 and explained how Tim-3 blockade using antibodies enhanced MHC-I-mediated antigen presentation in VSV encephalitis.

**Figure 7:**
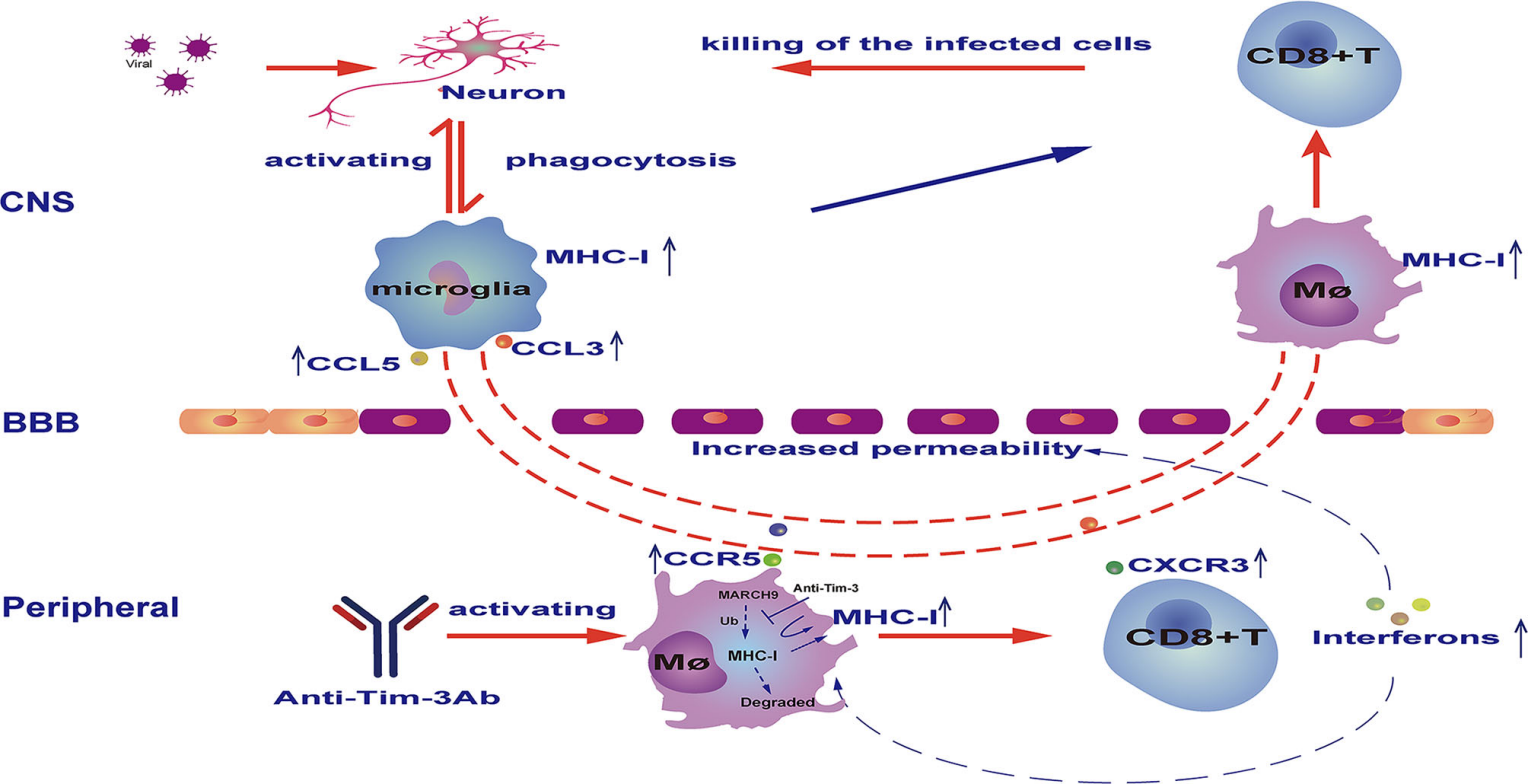
Schematic diagram of how Tim-3 antibody prevents vesicular stomatitis virus (VSV) encephalitis in mice. Peripheral injection of Tim-3 antibody promoted the activation of peripheral macrophages and CD8^+^ T cells, which was marked by an increased expression of chemokine receptors including C-C chemokine receptor type 5 (CCR5) and C-X-C chemokine receptor 3 (CXCR3), and increased expression of interferons. Notably, Tim-3 antibody enhanced major histocompatibility complex-I (MHC-I) expression by blocking Tim-3-mediated ubiquitination and degradation of MHC-I. As a consequence of cytokine and chemokine secretion, viral infection of the central nervous system (CNS) resulted in the recruitment of activated macrophages and CD8^+^ T cells into the brain, where the macrophages present the virus antigen and activate CD8^+^ T cells to cytotoxic T effector cells. BBB, blood-brain barrier.

A schematic diagram of how Tim-3 antibody augments peripheral immune cells to combat viral encephalitis is shown in **Figure 7**.

## Discussion

Viral encephalitis is a devastating disease with no specific treatment other than supportive care. Host immune responses play a critical role in fighting against the invading virus while maintaining neural homeostasis. Thus, methods that can be employed to appropriately manipulate the immune response during viral encephalitis are urgently needed. Here, we identified a novel mode of combating VSV encephalitis. Peripheral injection of Tim-3 antibody markedly decreased the viral load, tissue damage, and mortality associated with VSV encephalitis. Investigation of the underlying mechanism revealed that Tim-3 blockade augmented the systemic expression of interferons, enhanced the recruitment of macrophages and T cells into the brain, and increased the expression and antigen presentation activity of MHC-I on macrophages. Importantly, we observed a novel mechanism by which Tim-3 signaling regulated MHC-I expression; that is, by promoting proteasome-dependent degradation of MHC-I *via* E3 ligase MARCH9. Thus, our data shed light on the cross-talk between the peripheral immune response and CNS; consequently, we have discovered a novel mechanism that possibly augments their coordination to fight against viral encephalitis.

The cross-talk between the CNS and peripheral immune system under different physio-pathological conditions remains largely unclear. Neuroinflammation and neuroinfection can alter the BBB; however, whether this alteration is beneficial or not depends on different conditions. Further, there are controversial reports concerning whether the augmented immune response plays a protective or pathogenic role during neuroinfection and neuroinflammation (34). However, as there is no doubt that tightly regulated immune responses do play a critical role in infection control, it is urgent to identify ways to manipulate the immune response individually or to a proper state during viral encephalitis. In animal models of Japanese encephalitis, the BBB breaks down following infection, which results in a large influx of monocytes and lymphocytes into the brain (35, 36). Notably, the presence of CD8^+^ T cells and response of type I interferons are negatively correlated with the viral load; whereas, CD4^+^ T cells and innate immune responses are pathogenic (37). These studies provided possible targets, such as CD8^+^ T cells and interferons, that may be used to manipulate the immune response against viral encephalitis. Meanwhile, another study has shown that clodronate liposome-mediated depletion of monocytes/macrophages results in increased mortality, thereby indicating that hematogenously derived monocytes/macrophages are required for immune protection against viruses (38). These studies support our findings as they show that augmented recruitment of macrophages and T cells plays a protective role. During neuroinflammation, such as in Alzheimer’s disease, revitalizing brain immunity by recruiting peripheral myeloid cells and decreasing local immunosuppressive myeloid population is a novel therapeutic approach, which in turn supports the beneficial roles of the peripheral immune system (19). Thus, manipulating the immune response to a proper state is necessary for controlling different diseases. Here, we observed that enhanced CD8^+^ T cell activation following Tim-3 blockade played a protective role in virus control, and this finding is consistent with other studies that have demonstrated the protective role of CD8^+^ T cells in herpes simplex virus-1 encephalitis (39). The versatility of Tim-3 blockade in protection against other viral encephalitis will be investigated in future studies.

Here the antibody of Tim-3 is administrated before and after virus infection. We also tested the efficiency of anti-Tim-3 antibody on VSV encephalitis by injecting the antibody only after virus infection. The data in **Figure S6** showed that injecting the antibody only after virus infection could moderately, but not significantly, decrease the survival rate of the infected mice. However, it could significantly decrease the virus load and improve mice behavior in open field test, suggesting that injecting the antibody only after virus infection also provides protection against VSV encephalitis. Here we focus on exploring the mechanisms of anti-Tim-3 antibody injection before and after virus infection mediated protection on VSV encephalitis. Although the strategy in detail may be improved in the further, here we demonstrate the therapeutical potential of anti-Tim-3 antibody in virus encephalitis. VSV replication is very sensitive to type I interferons (IFN-I) signaling. As our data in **Figure S2** showed that anti-Tim-3 antibody increases the expression of interferons in mice *in vivo*, it is interesting to know whether anti-Tim-3 antibody mediated protection against VSV encephalitis depends on interferons. Our data in **Figure 3** showed that macrophages depletion with Clodronate Liposomes abolished Tim-3 antibody mediated protection. However, macrophages depletion with CL did not markedly alter the expression of interferons (**Figure S7**). These data suggested that the Tim-3 antibody mediated protection depends on macrophages rather than the increased interferons.

MHC-I-mediated antigen presentation and CD8^+^ T cell activation play critical roles in antiviral immunity. MHC-I expression can be regulated at both the mRNA and protein levels. In this study, we observed that peripheral injection of Tim-3 antibody systemically increased the expression of interferons, which possibly indirectly increases the transcription of MHC-I (31). Previously, we found that Tim-3 signaling may inhibit MHC-I transcription *via* STAT1 (40). Another study reported that viruses suppress MHC-I transcription *in vivo*, which might limit T cell-mediated killing of rotavirus-infected enterocytes (41, 42). Compared to the regulation at the transcriptional level, post-translational modification of MHC-I, which markedly affects the half-life of MHC-I, provides a more rapid control of antigen presentation (43). The ability of ubiquitin to control membrane protein localization and expression has significant consequences for cellular functions. Ubiquitination plays important regulatory roles in the immune system (44), and ubiquitination of MHC-I was first described in the context of viral proteins that target MHC-I for degradation in the endoplasmic reticulum and at the cell surface. Ubiquitination of MHC-I, MHC-II, and CD1a by different members of the MARCH family (32) of E3 ubiquitin ligases is a key event in the regulation of potent immunostimulatory properties of activated innate immune cells (45). Epstein-Barr virus inhibits MHC-I expression by regulating ubiquitination (46). Here, we identified a new post-translational mechanism of MHC-I that was mediated by Tim-3. By enhancing the expression of MARCH9, Tim-3 promoted the ubiquitination and degradation of MHC-I. These data explain the mechanism by which Tim-3 antibody injection enhanced MHC-I expression and antigen presentation in macrophages.

Recently, checkpoint inhibitors PD-1 and CTLA-4 have been used as therapeutic targets for immune disorders, tumors (47), autoimmune diseases (20) and neuroinflammatory diseases such as Alzheimer’s disease (19). Tim-3 is considered as the next-generation checkpoint inhibitor, and its antagonist has been widely investigated (48). We have previously focused on the role of Tim-3 in maintaining the homeostasis of innate immunity and found that Tim-3 inhibits TLR4-induced macrophage activation *via* the NF-κB signaling pathway (49), and inhibits M1 macrophage polarization *via* STAT1 (24). Macrophages also play critical roles in antiviral immunity both as an innate immune player and as antigen-presenting cells. In this study, we found that Tim-3 blockade using an antibody augmented the systemic immune response marked by increased expression of chemokines and their receptors, and as a result, enhanced the recruitment of infiltrating CD11b^+^CD45^hi^ pMDM into the brain, and more importantly, enhanced the antigen-presenting activity of the macrophages. The depletion of macrophages abolished Tim-3 antibody-mediated protection against VSV encephalitis, suggesting that macrophages play a critical role in Tim-3 blockade-mediated protection.

In summary, we identified a novel method that may be used to manipulate the immune response to combat viral encephalitis. Like PD-1, which can be used to attenuate neuroinflammation in Alzheimer’s disease by augmenting the systemic immune response, Tim-3 can be used to inhibit neuroinfection in VSV encephalitis by systemically augmenting the immune responses. Here, we identified that Tim-3 controlled the response of macrophages by enhancing the ubiquitination and degradation of MHC-I, and Tim-3 negatively regulated the macrophage-mediated antigen presentation. Our findings provide insight into the underlying mechanism of immune response against viral encephalitis and may have potential therapeutic applications.

## Data Availability Statement

The original contributions presented in the study are included in the article/**Supplementary Material**. Further inquiries can be directed to the corresponding authors.

## Ethics Statement

The animal study was reviewed and approved by Beijing Institute of Basic Medical Sciences.

## Author Contributions

Conception and design of the study: GH, GL, RW. Acquisition, analysis, and interpretation of data: GL, LT, CH, ZW, YG, JD. Contribution of administrative, experimental, analytic, or material support: SD, RM, YH, JD, JZ, BS. Writing-Original Draft Preparation: GL, RW. Writing-Review and Editing: GH. All authors contributed to the article and approved the submitted version.

## Funding

This work was supported by the National Natural Sciences Foundation of China (grant nos. 81971473, 81771684) and the Beijing Natural Sciences Foundation (grant no.7192145).

## Conflict of Interest

The authors declare that the research was conducted in the absence of any commercial or financial relationships that could be construed as a potential conflict of interest.

## Abbreviations

Tim-3, T- cell immunoglobulin and mucin domain-3; VSV, vesicular stomatitis virus; MHC-I, Major Histocompatibility Complex calss-1; pMDM, peripheral monocyte derived macrophages.
