# Supplementary material for: Peripheral Injection of Tim-3 Antibody Attenuates VSV Encephalitis by Enhancing MHC-I Presentation
Source: Front Immunol. 2021 May 7;12:667478. doi: 10.3389/fimmu.2021.667478 (PMC8138436; doi:10.3389/fimmu.2021.667478)
Supplement: Supplementary file 1 [file Table_1.docx]

**Supplementary Table and Figures**

**Table 1. Sequences of the primers used for PCR**

|  | Sense primer | Antisense primer |
| --- | --- | --- |
| 18s | 5‘-TTGACGGAAGGGCACCACCAG-3’ | 5‘-GCACCACCACCACGGAATCG-3’ |
| IFN-α | 5‘-TGCTGGCTGTGAGGACATAC-3’ | 5‘-AGGAAGAGAGGGCTCTCCAG-3’ |
| IFN-β | 5‘-CCCTATGGAGATGACGGAGA-3’ | 5‘-CCCAGTGCTGGAGAAATTGT-3’ |
| IFN-γ | 5‘-AAGCGTCATTGAATCACACCTG-3’ | 5‘-TGACCTCAAACTTGGCAATACTC-3’ |
| CCL3 | 5‘-CAAGTCTTCTCAGCGCCATATG-3’ | 5‘-CGTGGAATCTTCCGGCTGTA-3’ |
| CCL5 | 5‘-AAGTTCAGCTGCCCATCTG-3’ | 5‘-AGCATGCCTGGGTGGAAGT-3’ |
| MARCH9 | 5‘-GACGCGTGGGGCCTAATC-3’ | 5‘-GGTGAGTATGAGGCGGTGTC-3’ |

**Figure S1**

**
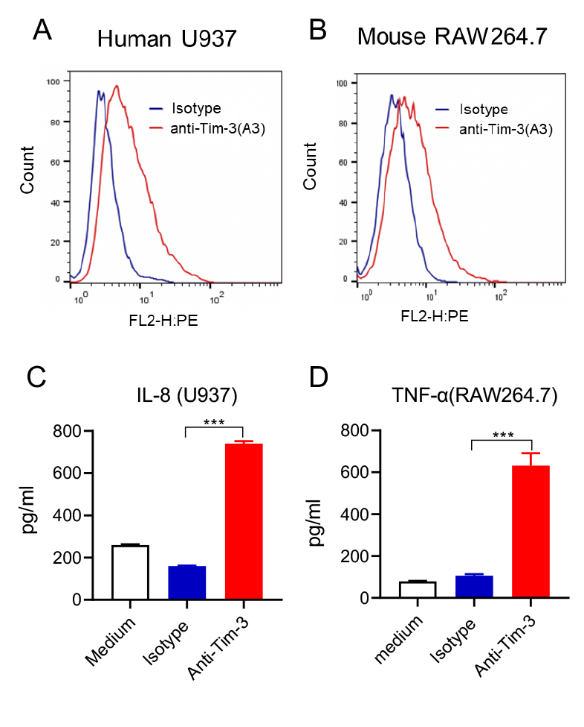
**

**Fig.S1: The binding and neutralization activity of anti-Tim-3 antibody (clone A3).**

(A&B) Human U937 cell line and mouse RAW264.7 cell line is incubated with anti-Tim-3 antibody (clone A3, 1ug/10^5^cells) respectively for 30min, using human IgG1 as isotype control, then after washing, PE-conjugated goat anti-human IgG antibody (1:1000) were added and incubated for another 30min. Finally, cells were washed and analyzed for staining by flow cytometry analysis. (C&D) Human U937 cell line and mouse RAW264.7 cell line is cultured in the presence of anti-Tim-3 antibody (clone A3, 10ug/ml) respectively for 24hours, using human IgG1 as isotype control. Then the supernatants were collected and analyzed for IL-8 and TNF-α production by ELISA. The data in A&B show the representative plot of three independent experiments. In C&D, the results are expressed as mean +SD of three independent experiments.

**Figure S2**

**
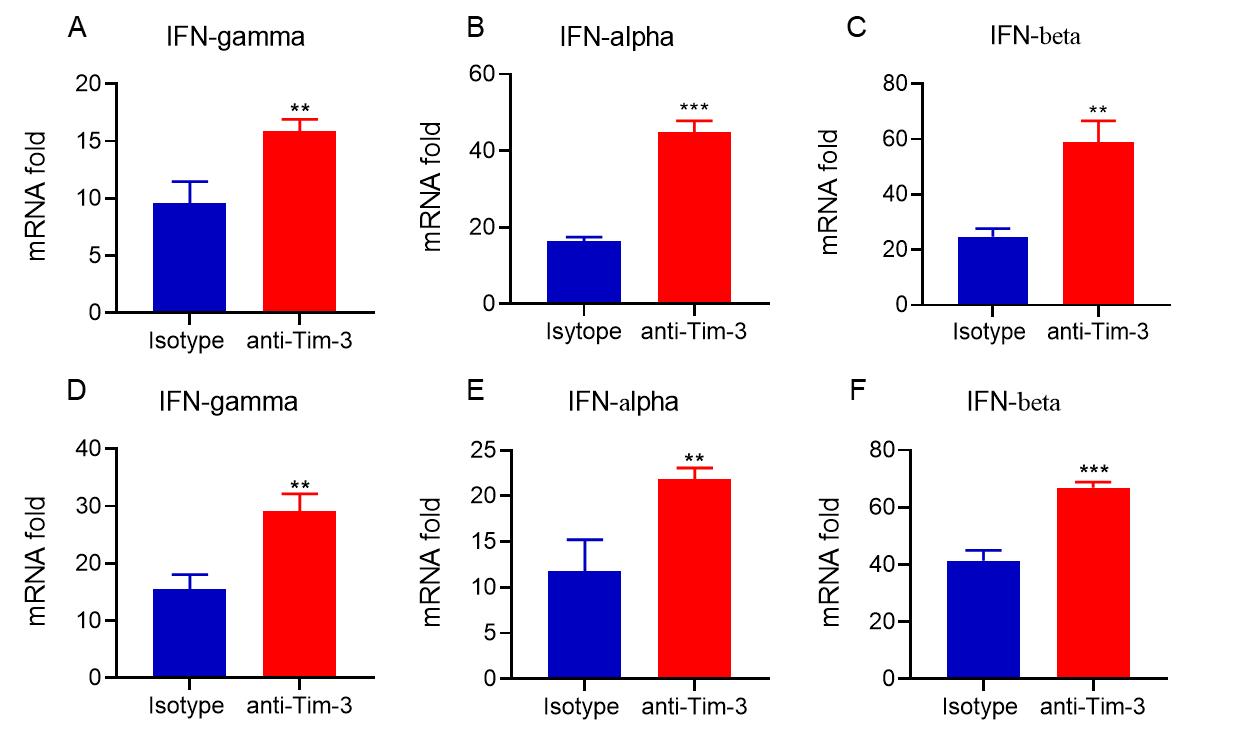
**

**Figure S2 Peripheral injection of Tim-3 antibody enhances the expression of interferons in vivo**

C57BL/6 mice were injected intraperitoneally with Tim-3 antibody (clone A3) (10mg/kg) or isotype control. Three days later mice were sacrificed and PBMC (A, B, C) and splenocytes (D, E, F) were collected and were analyzed for INF-alpha, IFN-beta and IFN-gamma expression by real-time polymerase chain reaction, and the results are expressed as mean ± SEM of three independent experiments. **, p<0.01, ***, p<0.001.

**Figure S3**

**
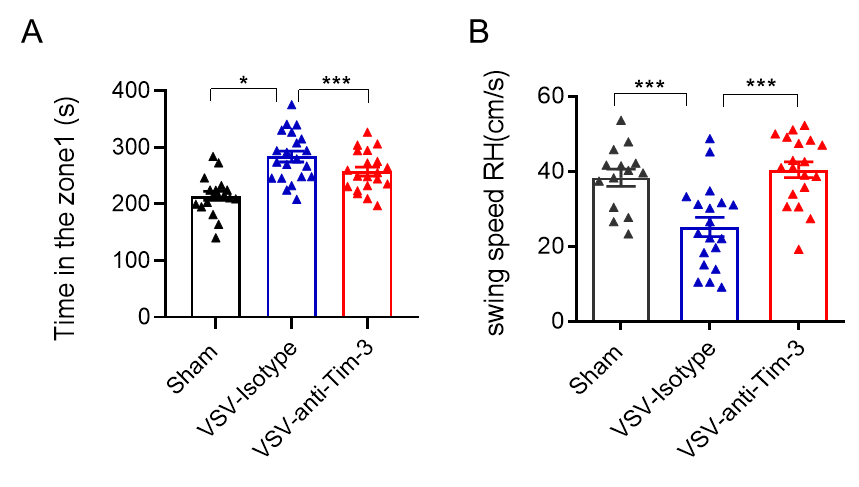
**

**Figure S3. Peripheral injection of Tim-3 antibody improves neuroethology in mice.** Mice were treated as in Fig.1. At the day 5 post infection, mice were submitted to the open field test to evaluate mice spontaneous locomotor activity through measurement of the Time in the zone1 for 10 min (A) and submitted to the CatWalk analysis to evaluate mice locomotor deficits through measurement of the swing speed for five trials per mouse with a maximum of 10s to traverse the glass plate (B). Untreated mice were used as sham control. Data are expressed as mean ± SEM of three independent experiments. *, p<0.05, ***, p<0.001.

**Figure S4**

**
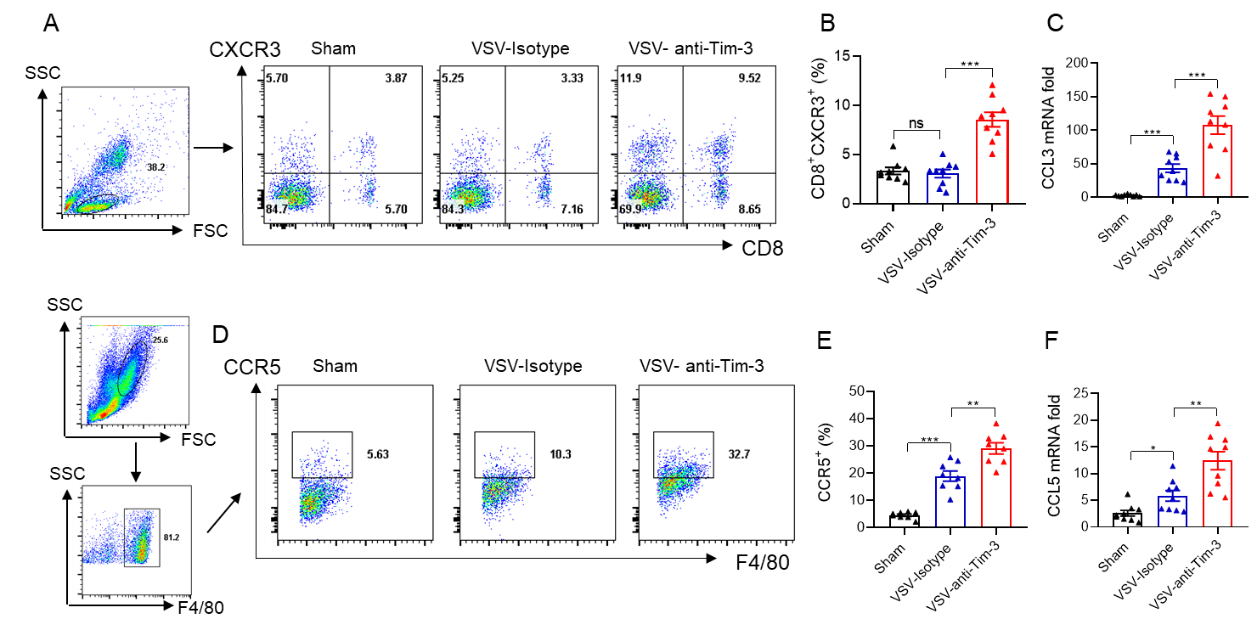
**

**Figure S4. Peripheral injection of Tim-3 antibody enhances the expression of chemokine receptor.** Mice were treated as in Fig.1. At day 5 post injection, mice were sacrificed. (A&B) PBMCs were used for the staining of CXCR3 on CD8^+^T cells by flow cytometry. A) shows the representative FACS dot , and B) shows the mean ± SEM of three independent experiments. (C) The brain tissues were collected to detect the expression of CCL3 by real-time PCR. The data are expressed as mean ± SEM of three independent experiments. (D&E) Peritoneal macrophages were used for the staining of CCR5 on F4/80^+^macrophage by flow cytometry. The representative FACS dot (D) and the mean ± SEM of three independent experiments were shown. (F) The brain tissues were collected to detect the expression of CCL5 by real-time PCR, and the results are expressed as mean ± SEM of three independent experiments. *, p<0.05, **, p<0.01, ***, p<0.001.

**Figure S5**

**
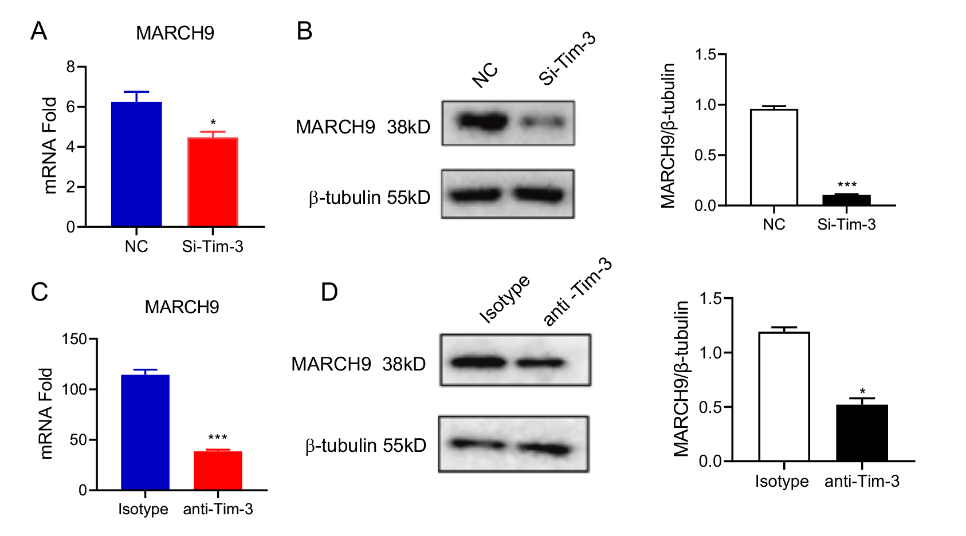
**

**Figure S5. Tim-3 inhibits MARCH-9 expression at both mRNA and protein level.** (A&B) RAW264.7 cells and si-Tim-3 RAW264.7 cells were analyzed for MARCH9 expression by real-time PCR (A) and Western blotting (B) analysis. (C&D) RAW264.7 cells were treated with isotype antibody or anti-Tim-3 antibody for 24 h, and then cells were collected for MARCH9 expression by real-time PCR (C) and Western blotting (D) analysis. In A&C， the data are expressed as mean ± SEM of three independent experiments. In B&D, left panels show the representative data of three independent experiments; right panels shows the mean ± SEM of three independent experiments. *, p<0.05, ***, p<0.001.

**Figure S6**

**
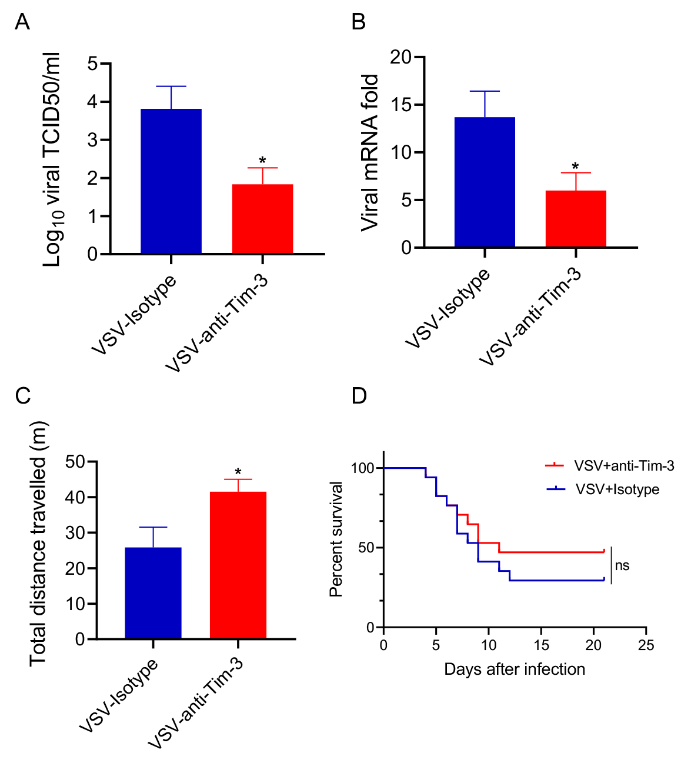
**

**Fig.S6. Effects of Tim-3 blockade post virus infection on VSV encephalitis**

C57BL/6 mice were intracranially injected with VSV (1×10^6^ pfu/g), and intraperitoneally injected with Tim-3 antibody (10mg/kg) or isotype control on days 1 and 2 post infection. (A&B) Brain tissues were collected on day 5 post-infection and VSV loads and virus replication were analyzed by TCID50 assay (A) and by real-time polymerase chain reaction (B) respectively. The results are expressed as mean ± SEM of three independent experiments. *, p<0.05. (C) At day 5 post infection, mice underwent the open-field test, and their spontaneous locomotor activity was evaluated by measuring the distance traveled in a defined area for 10 min. The results are expressed as mean ± SEM of three independent experiments. *, p<0.05. (D) The survival rate was analyzed. These experiment were repeated for two times with similar results.

**Figure S7.**


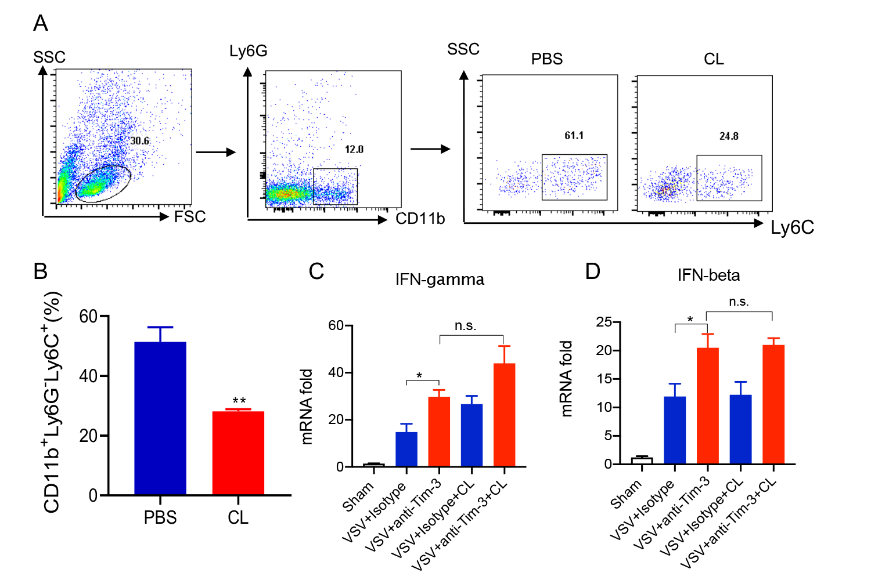


**Figure S7.** **Clodronate Liposomes mediated macrophages depletion and its effects on the expression of interferons**

Mice were treated as shown in Fig1, and intravenously injected Clodronate Liposomes (CL) as described previously in Fig.3. (A&B) The depletion efficiency of spleen macrophages (identified as CD11b^+^ Ly6G^-^Ly6C^+^) following CL injection was examined by flow cytometry analysis. A）shows the representative FACS plot of three independent experiments, B) shows the mean ± SEM of three independent experiments. (C&D) Brain tissues were collected on day 5 post-infection and were analyzed for IFN-gamma (C) and IFN-beta (D) expression by real-time polymerase chain reaction, and the results are expressed as mean ± SEM of three independent experiments. *, p<0.05, **, p<0.01.
